# Early-Onset Parkinson Disease Screening in Patients From Nigeria

**DOI:** 10.3389/fneur.2020.594927

**Published:** 2021-01-14

**Authors:** Lukasz M. Milanowski, Olajumoke Oshinaike, Benjamin J. Broadway, Jennifer A. Lindemann, Alexandra I. Soto-Beasley, Ronald L. Walton, Rana Hanna Al-Shaikh, Audrey J. Strongosky, Fabienne C. Fiesel, Owen A. Ross, Wolfdieter Springer, Shamsideen Abayomi Ogun, Zbigniew K. Wszolek

**Affiliations:** ^1^Department of Neurology, Mayo Clinic Florida, Jacksonville, FL, United States; ^2^Department of Neuroscience, Mayo Clinic Florida, Jacksonville, FL, United States; ^3^Department of Neurology, Lagos State University Teaching Hospital, Lagos, Nigeria; ^4^Neuroscience PhD Program, Mayo Clinic Graduate School of Biomedical Sciences, Jacksonville, FL, United States; ^5^Department of Clinical Genomics, Mayo Clinic Florida, Jacksonville, FL, United States

**Keywords:** Nigerian population, MLPA, Sanger sequencing, *LRRK2*, *PRKN*, *PINK1*, *DJ1*, Parkinson disease

## Abstract

**Introduction:** Nigeria is one of the most populated countries in the world; however, there is a scarcity of studies in patients with age-related neurodegenerative diseases, such as Parkinson disease (PD). The aim of this study was to screen patients with PD including a small cohort of early-onset PD (EOPD) cases from Nigeria for *PRKN, PINK1, DJ1, SNCA* multiplication, and LRRK2 p.G2019S.

**Methods:** We assembled a cohort of 109 Nigerian patients with PD from the four main Nigerian tribes: Yoruba, Igbo, Edo, and Hausa. Fifteen cases [14 from the Yoruba tribe (93.3%)] had EOPD (defined as age-at-onset <50 years). All patients with EOPD were sequenced for the coding regions of *PRKN, PINK1*, and *DJ1*. Exon dosage analysis was performed with a multiplex ligation-dependent probe amplification assay, which also included a *SNCA* probe and LRRK2 p.G2019S. We screened for LRRK2 p.G2019S in the entire PD cohort using a genotyping assay. The PINK1 p.R501Q functional analysis was conducted.

**Results:** In 15 patients with EOPD, 22 variants were observed [*PRKN*, 9 (40.9%); *PINK1*, 10 (45.5%); and *DJ1*, 3 (13.6%)]. Three (13.6%) rare, nonsynonymous variants were identified, but no homozygous or compound heterozygous carriers were found. No exonic rearrangements were present in the three genes, and no carriers of *SNCA* genomic multiplications or LRRK2 p.G2019S were identified. The PINK1 p.R501Q functional analysis revealed pathogenic loss of function.

**Conclusion:** More studies on age-related neurodegenerative diseases are needed in sub-Saharan African countries, including Nigeria. Population-specific variation may provide insight into the genes involved in PD in the local population but may also contribute to larger studiesperformed in White and Asian populations.

## Introduction

Sub-Saharan has one of the highest birth rates in the world. In 2019, the population Nigeria exceeded 200 million inhabitants, divided into 250 ethnic groups, with ~7 million Nigerians aged 65 years or older (1). The largest tribes in Nigeria are Hausa (30.0%), Yoruba (15.5%), and Igbo (15.2%) (2). The increasing number of aging Nigerians has prioritized studies evaluating the epidemiology and causes of Parkinson disease (PD), the prevalence of which is estimated at 10 to 235/100,000 people (3). However, there is still a lack of studies in this population. Most reports have concentrated on the prevalence of PD in Nigeria, environmental risk factors for PD in Nigeria, other diseases mimicking the clinical features of PD, and biochemical or pathological findings (3); The first Nigerian National PD Registry was just published in 2020 (4).

Genetic factors influence PD occurrence, especially in patients with positive family history or early-onset PD (EOPD; defined as age-at-onset <50 years) (5). In White populations, about 5–10% have monogenic forms of PD. The most common gene associated with PD is *LRRK2* (6). Missense mutation and multiplications have been reported in *SNCA* (7). *PRKN, PINK1*, and *DJ-1* are the three most common genes reported in EOPD (6). Functionally, PINK1 and PRKN protein together orchestrate the degradation of selectively damaged mitochondria via the autophagy-lysosome system, while DJ-1 operates in parallel to the PINK1-PRKN mitophagy pathway (8).

While most of these genes have been extensively examined only in White and Asian populations, three studies have included Nigerian patients for genetic analysis. Sanger sequencing was performed in *LRRK2, PRKN*, and *ATXN3* in 57 Nigerian patients with PD from Yoruba, Igbo, and Edo tribes (12.3% with EOPD) but did not identify any pathogenic mutations (9). The LRRK2 p.G2019S screening of 126 patients with PD was also negative (10). Fourteen Nigerian patients with PD were screened for 16 genes associated with PD. However exon dosage and *SNCA* multiplications analysis were never performed in this population (11).

There is also little data in the literature on PD in other sub-Saharan populations. In 39 Zambian patients with PD, a new potentially pathogenic mutation in LRRK2 p.A1464G and compound heterozygous mutations in *PRKN* were described (12). In a Ghanese study, no *LRRK2* variants were revealed (13). In a South African population, no LRRK2 p.G2019S mutations were identified in patients with African ancestry (14); however, in another study, two South African patients with EOPD had compound heterozygous mutations in *PRKN* (15).

Due to the lack of data on mutations in previously reported genes, analysis in the Nigerian population is warranted. We report data from the screening of apparently sporadic cases of PD from Nigeria [Yoruba (*n* = 86), Igbo (*n* = 2), Hausa(*n* = 19), and Edo (*n* = 2)] for *LRRK2* in all patients with PD and *PRKN, PINK1, DJ1*, and *SNCA* multiplications in patients with EOPD.

## Materials and Methods

**Figure 1:**
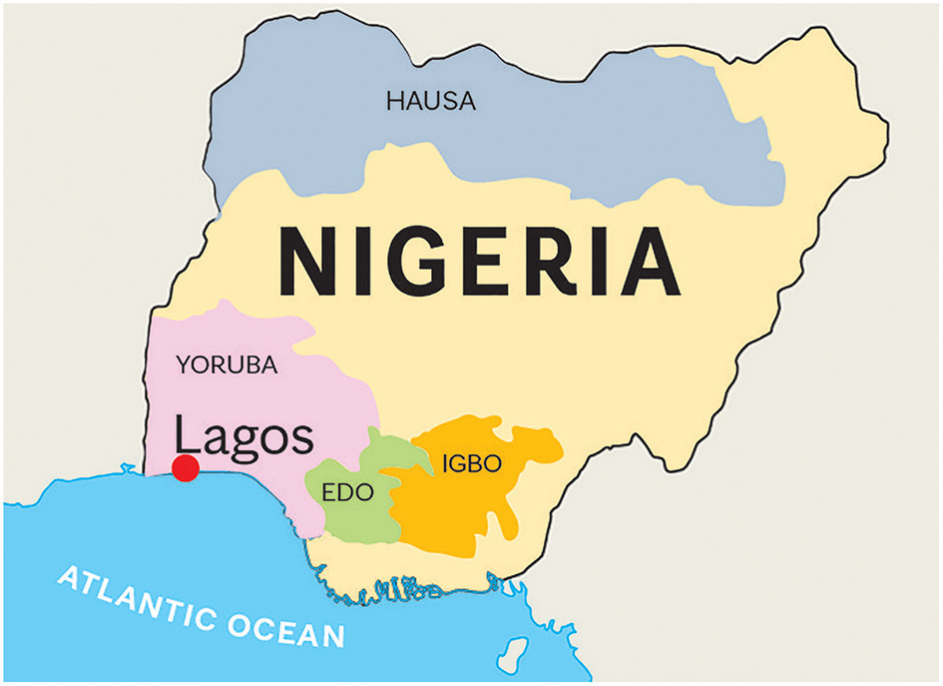
Map of Nigeria. Nigeria has 250 different tribes. We obtained blood samples from patients from Edo (*n* = 2), Hausa(*n* = 2), Igbo (*n* = 19), and Yoruba (*n* = 86) tribes. The estimated populations of these tribes are 5, 60, 30.4, and 31 million, respectively. Hausa is the largest Nigerian ethnic group that inhabits mostly the northern part of Nigeria. A large population lives also in the south of Niger. Yoruba tribe is located in the area of Lagos, the previous capital city of Nigeria. Igbo people inhabit the southern part of Nigeria, in the Biafra region. Edo people live in the Atlantic Ocean coastal areas. All samples were collected in Lagos, the largest port of Nigeria, with an estimated population of 8 million. The entire Nigerian population is over 200 million.

Blood specimens from a series of 109 clinical patients with PD were collected and characterized by movement disorder specialists (OO and SO) in the Division of Neurology at Lagos State University Teaching Hospital, Lagos, Nigeria. The study protocol was reviewed and approved by the Institutional Review Board of Lagos State University Teaching Hospital. Written informed consent for participation was not required for this study in accordance with the Nigerian national legislation and the institutional requirements. The Mayo Clinic IRB Committee approved this international collaboration. Although all patients were from Nigeria, their specific tribal origins were as follows: Yoruba, 86 (79.0%); Igbo, 19 (17.4%); Edo, 2 (1.8%); and Hausa, 2 (1.8%) (Figure 1). The collected blood specimens were then shipped to Mayo Clinic Florida in Jacksonville via international courier service. Diagnosis of PD was based on the UK Brain Bank diagnostic criteria for PD (16).

All patients with EOPD were Sanger sequenced for *PRKN* (exons 1–12), *PINK1* (exons 1–8), and *DJ1* (exons 1–6). Polymerase chain reaction products were purified using Mag-Bind TotalPure NGS and Mag-Bind SeqDTR chemistry (Omega Bio-tek, Inc) on the Biomek FX Automated Workstation (Beckman Coulter, Inc). Purified products were analyzed using a 3730*xl* DNA analyzer (Applied Biosystems), and sequences were analyzed using SeqScape Software v3.0 (Applied Biosystems). The identified variants were labeled according to appropriate reference sequences: *PRKN* (NM_004562), *PINK1* (NM_032409), and *DJ1* (NM_007262) (17). Mutations were referred to data from the Human Gene Mutation Database (18) and Genome Aggregation Database (gnomAD) (19). Multiple ligation-dependent probe amplification probemix P051 was used to screen for gene dosage (MRC Holland). This screening was performed using an 3730*xl* DNA analyzer, and the data were analyzed using Coffalyser. Net software (MRC Holland). The LRRK2 p.G2019S variant was also genotyped using TaqMan SNP Genotyping Assay (Applied Biosystems), and genetic analysis was completed using SDS2.2.2 software (Applied Biosystems). The potential pathogenicity of discovered variants was predicted with Combined Annotation Dependent Depletion (CADD) scores (CADD score >20).

For functional testing, *PINK1* cDNA was cloned into a V5-tagged expression vector (pcDNA6A *PINK1*-V5/His). The p.R501Q mutant was introduced by site-directed mutagenesis, and the presence of the mutation was confirmed by sequencing. PINK1 WT or p.R501Q were then expressed at or near-endogenous levels in previously established Hek293 PINK1 knockout (KO) cells (20). To mimic endogenous expression levels, 500 ng *PINK1*-V5 cDNA was diluted with 3,500 ng carrier DNA (pCMV-GST HA vector) and mixed with 5 μl Lipofectamine 2000 (Thermo Fisher). Cells were then transfected according to the manufacturer's instructions and were further treated the next day with 20 μM Carbonyl cyanide m-chlorophenyl hydrazine (CCCP) or with 10 μM MG132 (both Sigma) and harvested after 4 h. Cells were lysed in RIPA buffer with protease and phosphatase inhibitors (Roche). Protein (25 μg) was loaded onto 8–16% Tris-glycine gels, transferred onto PVDF (polyvinylidene fluoride) membranes, and probed with antibodies against PINK1 (#6946, Cell Signaling Technology, 1:2,000) and vinculin (V9131, Sigma, 1:100,000) as a loading control.

## Results

Of the 109 patients with PD, 15 had EOPD (13.8%; 14 from Yoruba and 1 from Igbo). In the whole study group, mean (SD) age of onset was 60.5 (9.1) years, and 77 participants (70.6%) were men. In the EOPD group, mean (SD) age of onset was 44.5 (5.0) years, and 10 participants (66.7%) were men. The cardinal symptoms of PD, such as bradykinesia, rigidity, and asymmetrical rest tremor, were observed in all patients. Postural instability was observed in 17 patients [15.6%; Yoruba, 14 (82.4%); Igbo, 2 (11.8%); and Edo, 1 (5.8%)]. There was no difference in postural instability occurrence between EOPD and late-onset PD [3 (20.0%) vs. 14/94 (14.9%); Fischer exact test, *P* = 0.70] (Table 1). One man with late-onset PD from Igbo had positive family history (0.9%). No LRRK2 p.G2019S mutation carriers were detected in our cohort (Table 1).

**Table 1:** *PINK1*,*DJ1*, and PRKN Variants in Nigerian Patients With Early-Onset Parkinson Disease (*n* = 15) and *LRRK2* G2019S in the Total Study Cohort (*N* = 109)^a^.

**rs number**	**AA**	**Alleles**	**Molecular consequences**	**Genotypes (major:het:minor)**	**MAF**	**MAF African (gnomAD)**	**MAF European (non-Finnish)** **(gnomAD)**	**CADD score**
*PINK1*								
rs537679886	E55E	c.165G>A	Synonymous	AA:AG:GG (14:1:0)	3.333%	0.59% (*n* = 77)	<0.01% (*n* = 1)	6.3
rs45530340	L63L	c.189C>T	Synonymous	CC:CT:TT (14:1:0)	3.333%	6.39% (*n* = 959)	19.91% (*n* = 13,063)	9.4
rs2298298		c.388-7A>G	Intronic	GG:AG:AA (8:7:0)	23.33%	76.22% (*n* = 18,979)	87.73% (*n* = 23,369)	2.7
rs142183624	L316L	c.948C>T	Synonymous	CC:CT:TT (13:2:0)	6.667%	1.29% (*n* = 321)	<0.01% (*n* = 3)	9.7
rs3131713		c.960-5G>A	Intronic	AA:AG:GG (7:8:0)	26.67%	76.50% (*n* = 18,991)	87.72% (*n* = 113,035)	5.8
**rs774946874**	**N410N**	**c.1230C>T**	**Synonymous**	**CC:CT:TT** **(14:1:0)**	**3.33%**	**0.00% (*****n*** **=** **0)**	**0.02%** **(*****n*** **=** **21)**	**15.2**
rs2298300		20516T>C	Intronic	CC:CT:TT (0:3:12)	90.00%	5.08% (*n* = 1,266)	0.09% (*n* = 109)	2.1
rs115477764	E476K	c.1426G>A	Missense	AA:AG:GG (14:1:0)	3.33%	4.00% (*n* = 999)	0.01% (*n* = 17)	14.3
rs61744200	R501Q	c.1502G>A	Missense	AA:AG:GG (14:1:0)	3.333%	3.25% (*n* = 811)	<0.01% (*n* = 811)	32
rs1043424	N521T	c.1562A>C	Missense	AA:AC:CC (8:7:0)	23.33%	26.45% (*n* = 6,596)	27.79% (*n* = 9,265)	14.2
*DJ1*								
rs11548933		c.-22 C>T	5′UTR	CC:CT:TT (7:7:1)	30.00%	13.22% (*n* = 2,933)	0.04% (*n* = 43)	2.9
rs11548937	G78G	c.234C>T	Synonymous	CC:CT:TT (11:4:0)	13.33%	11.13% (*n* = 2,765)	0.05% (*n* = 61)	11.5
rs72854882		c.323-14A>G	Intronic	AA:AG:GG (6:8:1)	33.33%	18.84% (*n* = 4,692)	0.08% (*n* = 98)	2.3
*PRKN*								
**rs112155221**		**c.-76-427G>A**	Intronic	**CC:CT:TT** **(14:1:0)**	**3.333%**	**0.0%** **(*****n*** **=** **0)**	**0.01%** **(*****n*** **=** **6)**	**11.5**
rs77795533	P37P	c.111G>A	Synonymous	AA:AG:GG (14:1:0)	3.333%	10.61% (*n* = 2,649)	0.04% (*n* = 56)	2.6
rs2075923		c.171+25T>C	Intronic	CC:CT:TT (10:5:0)	16.667%	38.88% (*n* = 9,681)	23.28% (*n* = 29,681)	5.5
rs1801474	S167N	c.500G>A	Missense	AA:AG:GG (9:6:0)	20.00%	7.17% (*n* = 7,807)	1.84% (*n* = 2,369)	15.7
rs9456735	M192L	c.574A>C	Missense	AA:AC:CC (13:2:0)	6.667%	5.94% (*n* = 1,483)	0.03% (*n* = 35)	20.7
rs9456711	L261L	c.783A>G	Synonymous	AA:AG:GG (9:6:0)	20.000%	17.79% (*n* = 4,464)	0.06% (*n* = 78)	7.7
rs114696251	Y267H	c.799T>C	Missense	TT:TC:CC (14:1:0)	3.333%	0.18% (*n* = 45)	<0.01% (*n* = 2)	27.8
rs144340740	G319G	c.957T>C	Synonymous	TT:TC:CC (14:1:0)	3.333%	3.45% (*n* = 859)	<0.01% (*n* = 7)	0.0
rs1801582	V380L	c.1138G>C	Missense	CC:CG:GG (8:4:3)	33.333%	17.48% (*n* = 4,359)	16.13% (*n* = 20,838)	11.2
***LRRK2***								
rs34637584	G2019S	c.6055G>A	Missense	GG:AG:AA (109:0)	0.000%	0.01% (*n* = 3)	0.03% (*n* = 33)	31

5′UTR, 5′ untranslated region; AA, amino acid; gnomAD, CADD-Combined Annotation Dependent Depletion; Genome Aggregation Database; Het, heterozygous; MAF, minor allele frequency.

a *Bold rows indicate variants not previously reported in African populations; gray rows, variants found more often in African populations than in European (non-Finnish) in gnomAD*.

In 15 patients with EOPD, 22 variants were discovered in three genes [*PRKN*, 9 (40.9%); *PINK1*, 10 (45.5%); and *DJ1*, 3 (13.6%)]. In all genes, there were six intronic variants (27.3%), one in 5′ untranslated region (4.5%), eight coding synonymous (36.4%), and seven coding non-synonymous (31.8%). rs774946874 in *PINK1* and rs112155221 in *PRKN* have not been observed in African populations but have been reported in European (non-Finnish) ancestries. Two variants in *PRKN*, three in *DJ1*, and three in *PINK1* occur more often in African than in European (non-Finnish) populations in gnomAD. We found three (13.6%; two from Yoruba and one from Igbo) rare, non-synonymous variants (defined as minor allele frequency <5% in gnomAD), but no homozygous or compound heterozygous carriers were present. No exonic rearrangements were observed (Table 1).

**Figure 2:**
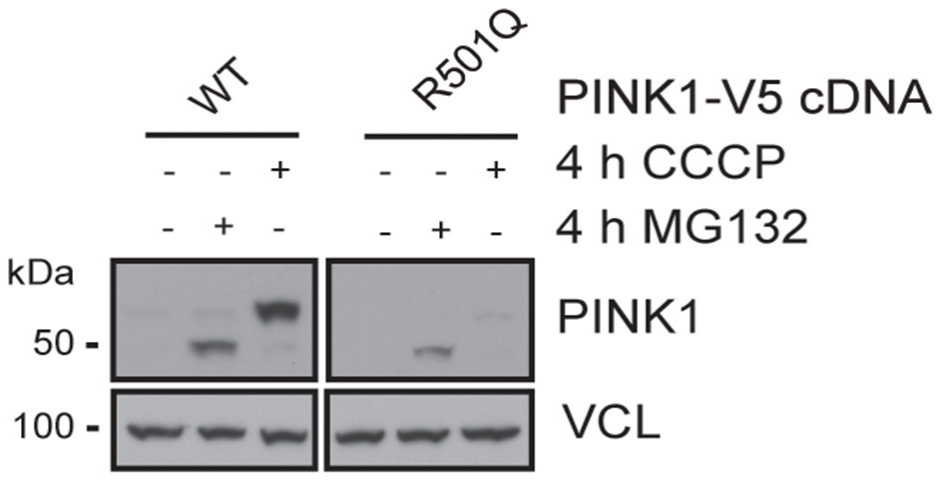
PINK1 p.R501Q is unstable and poorly accumulates on the outer mitochondrial membrane upon stress. PINK1 KO Hek293 cells were transfected with *PINK1*-V5 tagged cDNA using special conditions to mimic endogenous expression levels, and protein lysates were analyzed by western blot. PINK1 WT is almost undetectable at basal conditions but is swiftly stabilized as a full-length protein (~63 kDa) following mitochondrial damage (CCCP treatment). However, the PINK1 p.R501Q mutant remains unstable and only poorly accumulates on the outer mitochondrial membrane upon stress. Yet, the cleaved forms of PINK1 WT and p.R501Q (~52 kDa) can be stabilized in the cytosol upon proteasome inhibition (MG132 treatment), confirming the transient expression of both variants.

PINK1 and PRKN together orchestrate a stress-induced mitochondrial quality control pathway that can be probed at multiple steps along its sequence to functionally assess the pathogenicity of genetic variants (8). While both *PRKN* variants have been analyzed earlier (21, 22), to our knowledge the pathogenicity of the *PINK1* variant has never been tested before. As part of its surveillance, PINK1 WT is constitutively imported into healthy mitochondria, where it is N-terminally cleaved, exported to the cytosol, and degraded by the proteasome. Upon mitochondrial damage, PINK1 can no longer be imported and thus locally accumulates as a full-length protein on the outer mitochondrial membrane where it initiates mitophagy through the activation and recruitment of PRKN. To assess the functionality of the identified variant, PINK1 KO Hek293 cells were transfected with either WT or p.R501Q mutant *PINK1* cDNA (Figure 2). Using conditions that result in near-endogenous expression levels, full-length PINK1 WT was only detectable upon mitochondrial depolarization (4 h CCCP), whereas PINK1 p.R501Q appeared highly unstable and only poorly accumulated even following CCCP treatment. Transient, though successful, expression of either *PINK1* variant in cells was confirmed after proteasome inhibition (4 h MG132) which in both cases stabilized the N-terminally cleaved form of PINK1.

## Discussion

Genetic studies of PD in sub-Saharan African countries are sparse. We comprehensively screened a small (*n* = 15) series of patients with EOPD from Nigeria for the most commonly associated genes (*PRKN, PINK1*, and *DJ1, SNCA* multiplication) and screened a larger series (*n* = 109) of Nigerian patients with PD for LRRK2 p.G2019S. No pathogenic mutations were revealed. Two observed variants have been found previously only in non-African populations, and 15 have been reported more often in African than European (non-Finnish) populations in gnomAD. In the EOPD group, seven discovered variants were non-synonymous coding variants.

Clinical characteristics in the analyzed patients were consistent with typical PD symptoms in other populations. *LRRK2* variants are commonly found in the Mediterranean area and northern African countries. However, similar to other sub-Saharan African study groups, no LRRK2 p.G2019S variants were detected (9, 10, 23). The percentage of patients with EOPD in our cohort was similar to previous reports (9, 10). There were no homozygous or compound heterozygous EOPD mutations carriers in genes causing autosomal recessive EOPD.

In a previous study of Nigerian patients with PD, only *PRKN* was sequenced, with 10 variants reported, but no pathogenic mutations (24). In our study, two potential pathogenic heterozygous substitutions were discovered in PRKN (p.M192L, CADD score = 20.7 and p.Y267H, CADD score = 27.8) and one in PINK1 (p.R501Q, CADD score = 32). PRKN p.M192L and p.Y267H have been reported in a previous Nigerian study, which analyzed *PRKN* mutations in Yoruba, Igbo, and Edo tribes (9). Both variants are most frequently reported in Black African populations in gnomAD.

The herein identified PRKN mutants p.M192L and p.Y267H had been previously analyzed in cell-based mitophagy paradigms and using different functional readouts. No obvious defect was found for PRKN p.M192L (or p.M192V), and as such this variant was functionally classified as benign (21). However, PRKN p.Y267H showed an early delay in translocation to damaged mitochondria compared to PRKN WT but perhaps more importantly a significant reduction in ubiquitin charging of its active site (22). This defect is reflective of overall reduced enzymatic activity and thus is supportive of a pathogenic *PRKN* loss of function.

Similarly, the PINK1 p.R501Q variant that we functionally tested here likely results in a pathogenic loss of function. Compared to PINK1 WT, p.R501Q was unstable and only very poorly accumulated upon stress on the outer mitochondrial membrane. Although we have used special conditions to mimic near-endogenous expression, the results are based on transient transfections and as such need to be verified. However, we recently identified another PINK1 variant (p.I368N) with a similar phenotype that was unstable as a full-length protein but could be stabilized as a cleaved form upon proteasome inhibition in patients' fibroblasts (25).

To our knowledge, this is the first study in which multiple ligation-dependent probe amplification was performed in a Nigerian population and the first time patients from Nigeria's largest tribe, Hausa, were screened for LRRK2 p.G2019S. Although we did not find any exonic rearrangements, they have been discovered in another sub-Saharan population (15). In White and Asian patients, 43.2% of *PRKN* mutations may be structural variants (26). These data suggest that exon dosage analysis should always be performed in potential *PRKN* mutation carriers.

Our study has several limitations. Our small study cohort may not reflect the prevalence of reported PD genes in all tribes analyzed. We also had limited clinical characteristics for our study population. Genetic factors are usually present in populations with EOPD or family history of PD, so including these groups into analysis increases the chance of reporting positive results (27).

Further analyses are urgently needed to characterize the genetic variation in Nigeria. Our study is the first step in genetic characterization of known PD genes in four tribes in Nigeria. Future studies should include larger cohorts with better clinical characterization. Known genes should be analyzed first, then a genome-wide association study on a population of non-carriers may lead to discovery of unique loci responsible for PD in sub-Saharan Africans.

## Data Availability Statement

The original contributions presented in the study are included in the article/supplementary material, furtherinquiries can be directed to the corresponding author/s.

## Ethics Statement

The studiesinvolving human participants were reviewed and approved by the Institutional Review Board of Lagos State University Teaching Hospital. Written informed consent for participation was not required for this study in accordance with the Nigerian national legislation and the Nigerian institutional requirements. The Mayo Clinic IRB Committee approved this international collaboration.

## Author Contributions

LM contributed to analysis and interpretation of the data, drafting of the article, and generation/collection of images. OO and SO contributed to conception and design, collection, analysis, and interpretation of the data, and critical revision of the article. BB performed the functional experiments and contributed to the analysis and interpretation of the data, drafting and critical revision of the article, and generation of images. JL, AB, and RW contributed to experiments, analysis and interpretation of the data, and critical revision of the article. RH and AS contributed to analysis and interpretation of data and critical revision of the article. FF and WS contributed to conception and design, analysis and interpretation of the data, drafting and critical revision of the article, and generation of images. OR contributed to conception and design, analysis and interpretation of the data, and drafting and critical revision of the article. ZW contributed to conception and design, collection, analysis, and interpretation of the data, drafting and critical revision of the article, and generation/collection of images. All authors approved the final article.

## Conflict of Interest

The authors declare that the research was conducted in the absence of any commercial or financial relationships that could be construed as a potentialconflict of interest. The handling Editor declared a past co-authorship with several of the authors OR, ZW at time of review.
